# Discovery of a novel whitefly- and aphid-transmitted polerovirus on rice plants with dwarfing and fewer tillering symptoms

**DOI:** 10.1007/s44297-024-00033-0

**Published:** 2024-08-06

**Authors:** Jiaping Yu, Ming Zeng, Yali Zhou, Jirui Wang, Xueping Zhou, Jianxiang Wu

**Affiliations:** 1 State Key Laboratory of Rice Biology, Key Laboratory of Biology of Crop Pathogens and Insects of Zhejiang Province, Institute of Biotechnology, Zhejiang University, Hangzhou, 310058 China; 2 Hainan Institute, Zhejiang University, Sanya, 572025 China; 3 College of Advanced Agricultural Sciences & Key Lab for Biology of Crop Pathogens and Insect Pests and Their Ecological Regulation of Zhejiang Province, Zhejiang Agriculture & Forestry University, Linan, 311300 China; 4 State Key Laboratory for Biology of Plant Diseases and Insect Pests, Institute of Plant Protection, Chinese Academy of Agricultural Sciences, Beijing, 100193 China

**Keywords:** Novel polerovirus, Rice, Rice dwarf polerovirus, Rice whitefly *Bemisia formosana* Takahashi

## Abstract

Rice is the most important cereal grain worldwide and is cultivated mainly in Asian countries. Rice viral disease epidemics caused by insect-borne viruses are a major threat to rice production. However, to date, how many unknown viruses naturally infect rice plants in the field remains vague. Here, we identified a novel polerovirus tentatively named rice dwarf polerovirus (RDPV) via RNA-seq technology in rice plants showing dwarfing and fewer tillering symptoms collected from paddy fields in Hainan Province, China. The RDPV genome consists of a positive-sense, single-stranded RNA molecule that is 5832 nt in size and is deduced to encode seven viral proteins. Phylogenetic analyses based on the full-length genome sequence and RNA-dependent RNA polymerase (RdRp) amino acid sequence revealed that RDPV is in an evolutionary branch with known viruses in the *Polerovirus* genus. Interestingly, virus transmission experiments demonstrated that RDPV can be transmitted by two completely different vectors, i.e., whiteflies and aphids, from diseased rice plants to healthy rice seedlings. Furthermore, we constructed an infectious cDNA clone of RDPV and found that it can infect *Nicotiana benthamiana* plants via Agrobacterium-infiltrated inoculation. Thus, we concluded that RDPV is a novel whitefly- and aphid-transmitted rice polerovirus. These findings contribute to expanding our knowledge of rice polerovirus diversity, and developing efficient strategies to manage this rice viral disease.

**Supplementary Information:**

The online version contains supplementary material available at 10.1007/s44297-024-00033-0.

## Introduction

According to the International Committee on the Taxonomy of Viruses (ICTV, http://ictv.global/report), members of the family *Solemoviridae* are now classified into four genera: *Sobemovirus*, *Polemovirus*, *Polerovirus* and *Enamovirus*. Poleroviruses have a polycistronic, positive-sense, single-stranded RNA genome with a viral protein genome-linked (VPg) covalently attached to the 5’ terminus of the genome and no poly(A) tail at the 3’ end, which often contains seven open reading frames (ORFs) [1, 2]. The viral proteins of poleroviruses are expressed through genomic and subgenomic RNA. The viral proteins P0, P1 and RdRp are expressed from genomic RNA, whereas P3a, P3, P4 and P5 are encoded by subgenomic RNA1, and both ORF6 and ORF7 are translated from subgenomic RNA2 [3]. The P0 protein functions as a viral suppressor of RNA silencing [4]. RdRp is a translational fusion protein produced by ORF1 and ORF2 through a -1 ribosomal frameshift mechanism (http://ictv.global/report). P3a is involved in long-distance viral movement and phloem-restricting [3, 5]. P4 functions as a movement protein required for viral cell-to-cell movement [6]. P5 plays an essential role in virus infection [7]. Polerovirus infection is characterized by phloem necrosis, leaf streaking, yellowing, rolling and thickening, fruit discoloration and plant dwarfing [8] (ICTV, http://ictv.global/report). Phloem-limited poleroviruses are transmitted exclusively by aphids in a persistent, circulative and non-propagative manner [8, 9].

With the rapid development of high-throughput RNA-sequencing (RNA-seq), many novel viruses have been discovered in recent years [10, 11]. For example, several novel plant viruses of rice curl dwarf-associated picornavirus [12], paederia scandens chlorosis yellow virus [13], rice dwarf-associated bunya-like virus [11], and rice-associated noda-like viruses 1 and 2 [14] were discovered in our previous works. In recent years, Yan et al. revealed that rice tiller inhibition virus (RTIV), a novel aphid-transmitted polerovirus in Asian wild rice plants, can cause low-tillering disease in rice cultivars after aphid transmission [9].

Rice is one of the most important cereal grains worldwide, with an annual global production of 650 million tons, more than 90% of which are produced in Asian countries. However, rice constantly experiences outbreaks of known and unidentified viral diseases, resulting in significant yield and economic losses. In China, there are currently eight main rice viruses, that is, southern rice black-streaked dwarf virus, rice black-streaked dwarf virus, rice stripe virus, rice ragged stunt virus, rice dwarf virus, rice gall dwarf virus, rice grassy stunt virus, and rice stripe mosaic virus. These viruses are transmitted by planthoppers or leafhoppers and cause severe damage to rice production [14]. In this work, we identified a novel polerovirus tentatively named rice dwarf polerovirus (RDPV) via RNA-seq in rice plants with dwarfing and fewer tillering symptoms collected from paddy fields in Hainan Province, China. RDPV can be transmitted by whiteflies and aphids. Furthermore, we constructed an infectious cDNA clone of RDPV and found that it can infect *Nicotiana benthamiana* via agrobacterium-mediated infiltration. The findings of this work shed light on the discovery of rice viruses and can be used to develop efficient strategies for managing this rice viral disease.

## Results

### Identification of a novel polerovius in rice plants

**Fig. 1:**
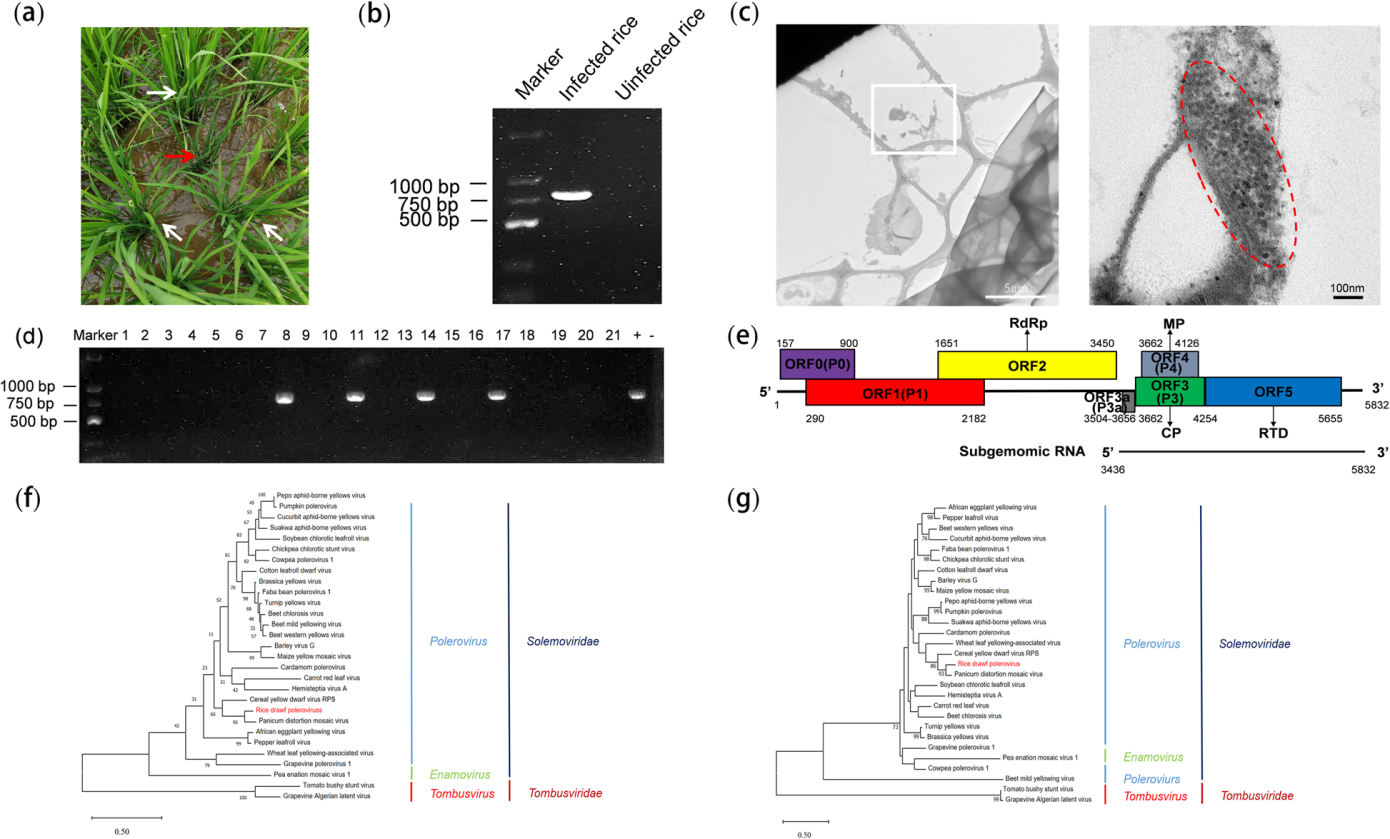
Discovery of rice dwarf polerovirus (RDPV). **a** Dwarfing and less tillering symptoms of RDPV-infected rice plants in a rice field in Hainan Province of China. The red arrow points to an infected rice plant, and the white arrows point to uninfected rice plants. **b** RT‒PCR detection of RDPV infection in the rice plants shown in (**a**) via specific primer pairs designed according to the assembled contig. **c** Transmission electron microscopy image of RDPV virions in vascular bundle cells of infected plant tissues. The image on the right is an enlargement of the boxed area on the left. **d** RT‒PCR detection of RDPV infection in field-collected rice plants. Lanes 1–21 represent 21 field rice plant samples with dwarf symptoms collected from paddy fields in Hainan Province, China. + represents an RDPV-infected rice plant used as the positive control, whereas - represents an uninfected rice plant used as the negative control. **e** Schematic diagram of the RDPV genome organization drawn in a size ratio column. **f**-**g** Phylogenetic trees of RDPV were constructed on the basis of the amino acid sequences of RdRp (**f**) and CP (**g**) via the maximum likelihood method with 1000 bootstraps. The bootstrap values are indicated adjacent to the nodes. The RDPV is shown in red. The other known viruses used in this study are listed in Table S2

In April 2023, some rice plants with symptoms of dwarfism and fewer tillers were discovered in rice fields in Sanya City, Hainan Province, China, and were collected to identify viral pathogen(s) via RNA-seq (Fig. 1a). After RNA-seq and filtering the low-quality reads, we utilized Trinity v2.3.2 software to assemble the resulting clean reads de novo into a long contig of 5,796 nucleotides (nts). The RT‒PCR results obtained via the use of contig-specific primer pairs (Table S1) further demonstrated that this contig indeed existed in the collected rice samples (Fig. 1b). BLASTx searches of the NCBI database revealed that this contig query has amino acid (aa) sequence homology with members of the genus *Polerovirus* of the family *Solemoviridae*, which indicates the presence of a putative novel virus tentatively named rice dwarf polerovirus (RDPV) in these rice plants. We then performed 5’ and 3’ rapid amplification of cDNA ends (5’/3’ RACE) to determine the viral 5’ and 3’ terminal sequences. The 5’/3’ RACE and RT‒PCR results revealed that the complete genome of RDPV is 5832 nt in length, which is now deposited in the NCBI GenBank under accession number PP925870. Poleroviruses have the typical characteristics of vascular bundle localization [15, 16]. To determine whether RDPV is located in vascular bundle cells, RDPV-infected plant tissues were investigated via transmission electron microscopy (TEM), and TEM revealed that the infected plant tissues contained numerous spherical virus particles approximately 30 nm in diameter clustered in the cytoplasm of vascular bundle cells (Fig. 1c), similar to the morphology and location of known poleroviruses. Taken together, the data above indicate that the collected dwarf rice plants were infected by RDPV.

To determine the incidence rate of RDPV in paddy fields in Hainan Province, China, 21 field samples showing dwarf symptoms were collected from different paddies in Sanya city and Lingshui County in April 2024, and RDPV infection was investigated via RT‒PCR. The RT‒PCR results revealed that 4 of the 21 samples were RDPV positive (Fig. 1d), suggesting that RDPV is epidemic in rice fields in Hainan Province, China.

### Genome organization and viral proteins of RDPV

RDPV has a positive-sense, single-stranded RNA genome containing a 177-nt 3’ untranslated region (UTR) and a 156-nt 5’ UTR with a conserved ‘ACAAAAGA’ motif, similar to other known poleroviruses which are covalently attached to a VPg. Like other poleroviruses, the RDPV genome was deduced to encode three viral proteins (P0, P1 and RdRp), whereas its subgenome was deduced to encode four proteins (P3a, P3, P4 and RTD) (Fig. 1e) [2, 17].

Sequence alignment analysis revealed that RDPV shares 45.7–77.9% genome sequence identity with other known viruses in the genus *Polerovirus* and shares the highest identity with panicum distortion mosaic virus (PDMV) (Table S3). ORF0 was deduced to encode a putative RNA silencing suppressor protein (P0) [18], which shares the highest amino acid (aa) sequence identity (62.8%) with PDMV (Table S3). ORF1 (nt positions 290–2182) encodes a polyprotein (P1), which shares 10.3%–70.2% aa sequence identity with other known poleroviruses (Table S3). ORF1 and ORF2 (nt positions 290–2182, 1651–3450) were speculated to yield a fusion protein, putative RdRp, via a -1 ribosomal frameshift, which contains the conserved domain ps-ssRNAv_RdRp-like superfamily (cl40470, 580-1033 aa). RdRp shares 19.6%–78.6% aa sequence identity with known poleroviruses, with the highest identity with PDMV (Table S3). The putative coat protein (CP), also known as P3, is encoded by ORF3 (nt positions 3628-4245) and contains a Luteo_coat superfamily domain (cl03007, 69-204 aa). The aa sequence identity of RDPV P3 with known viruses in  the genus *Polerovirus* ranges from 43.2% to 87.8% (Table S3). There is a 1063-nt IGR between the translation boxes of RdRp and CP, and we speculated that the non-AUG-initiated protein P3a starts at nt position 3495 and ends at a UAG stop codon at nt position 3647 within this region. According to previous reports [3], RDPV P3a may play a role in long-distance viral movement. ORF4 (nt positions 3662-4126) overlapping ORF3 was deduced to encode a viral movement protein (MP) also known as P4, which has the highest aa identity of 87.6% with PDMV (Table S3). ORF5 (nt positions 4255–5655) is translated via an in-frame readthrough of the ORF3 stop codon to encode a 467 aa minor capsid, also known as the readthrough protein (RTP). RDPV RTD shares 25.1–76.1% aa identity with other known poleroviruses (Table S3). The species demarcation criterion in the genus *Polerovirus* is that differences in the aa sequence identity of any viral gene product are greater than 10% [2]. On the basis of the above findings, we concluded that RDPV is a new species in the genus *Polerovirus*.

### Evolutionary relationship of RDPV

To elucidate the evolutionary relationship of RDPV, phylogenetic trees were constructed using the RdRp and CP aa sequences of 28 related known viruses (Table S2) and RDPV (Fig. 1f and g). The resulting trees revealed that RDPV falls on the same branch as other known viruses from the genus *Polerovirus* of the family *Solemoviridae* and is most closely related to PDMV, an unassigned polerovirus (Fig. 1f and g). This result is in line with the results of the viral protein identity analyses (Table S2).

### RDPV is transmitted by both rice whitefly and aphid vectors

**Fig. 2:**
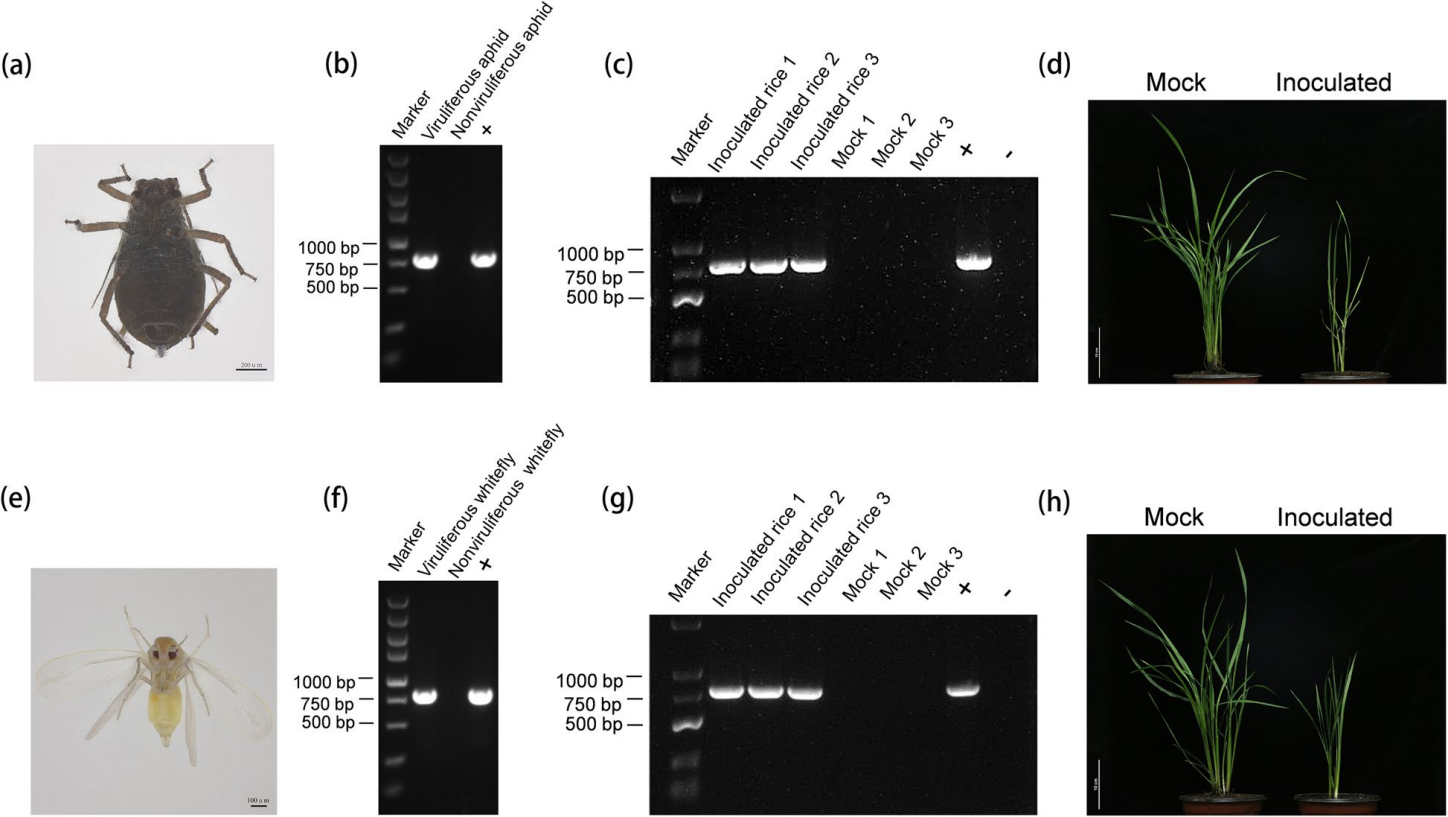
RDPV is transmitted by both rice whitefly and aphid vectors. **a ***R. padi* nymph. **b** RT‒PCR results of RDPV infection of *R. padi* fed RDPV-infected rice plants for 3 days. + represents an RDPV-infected rice plant used as the positive control, and nonviruliferous *R. padi* that fed on uninfected rice plants for 3 days were used as the negative control. **c** RT‒PCR results of inoculated rice plants fed viruliferous *R. padi* at 14 days post inoculation (dpi). Mocks 1–3 included three rice plants fed nonviruliferous *R. padi*. + represents an RDPV-infected rice plant used as the positive control, whereas - represents an uninfected rice plant used as the negative control. **d** Symptoms of an RDPV-infected rice plant (right) fed by viruliferous *R. padi* at 28 dpi. The mock treatment consisted of a rice plant (left) fed nonviruliferous *R. padi*. **e ***Bemisia formosana* Takahashi adult. **f** RT‒PCR results of RDPV infection of *B. formosana* Takahashi fed on RDPV-infected rice plants for 3 days. + RDPV-infected rice plants used as the positive control and nonviruliferous *B. formosana* fed on uninfected rice plants for 3 days were used as the negative control. **g** RT‒PCR results of inoculated rice plants fed viruliferous *B. formosana* Takahashi at 14 dpi. Mocks 1–3 included three rice plants fed by nonviruliferous *B. formosana* Takahashi. + represents an RDPV-infected rice plant used as the positive control, whereas - represents an uninfected rice plant used as the negative control. **h** Symptoms of an RDPV-infected rice plant (right) fed by viruliferous *B. formosana* Takahashi at 28 dpi. The mock treatment consisted of a rice plant (left) fed by nonviruliferous *B. formosana* Takahashi

Poleroviruses are exclusively transmitted by aphids [19]. In addition, a recent study reported that aphid can transmit a polerovirus RTIV discovered on wild rice plants [9]. Notably, we found a rice whitefly (*Bemisia formosana* Takahashi) on RDPV-infected rice plants in the field. Furthermore, Ghosh et al. [8] reported a new pepper polerovirus transmitted by a new whitefly. Thus, we speculate that this rice whitefly may be an insect vector of RDPV. To investigate which insect vector can transmit RDPV, the virus-free aphids *Rhopalosiphum padi* and *Sitobion avenae* and *Schizaphis graminum* and *B. formosana* Takahash were fed on RDPV-infected rice plants for 3 days and then transferred to healthy rice seedlings for feeding for another 7 days. All the analyzed vectors were subjected to RT‒PCR detection to investigate the presence of RDPV, and the results demonstrated that *R. padi* (Fig. 2a and b) and *B. formosana* Takahash (Fig. 2e and f) can obtain RDPV, but both *S. avenae* and *S. graminum* cannot (Fig. S1). Furthermore, the RT‒PCR results revealed that the rice seedlings fed with viruliferous *R. padi* (Fig. 2c) or viruliferous *B. formosana* Takahash (Fig. 2g) were infected with RDPV 14 days post inoculation (dpi). At 28 dpi, the rice plants fed with viruliferous *R. padi* (Fig. 2d) or viruliferous *B. formosana* Takahash (Fig. 2h) displayed symptoms of dwarfing and fewer tillers. The above findings indicate that both *R. padi* and *B. formosana* Takahash can transmit RDPV from diseased rice plants to healthy rice seedlings and are two insect vectors of RDPV. Surprisingly, this is the first time that a polerovirus can be transmitted by two completely different insect vectors, i.e., whiteflies and aphids.

### An infectious cDNA clone of RDPV and its inoculation

**Fig. 3:**
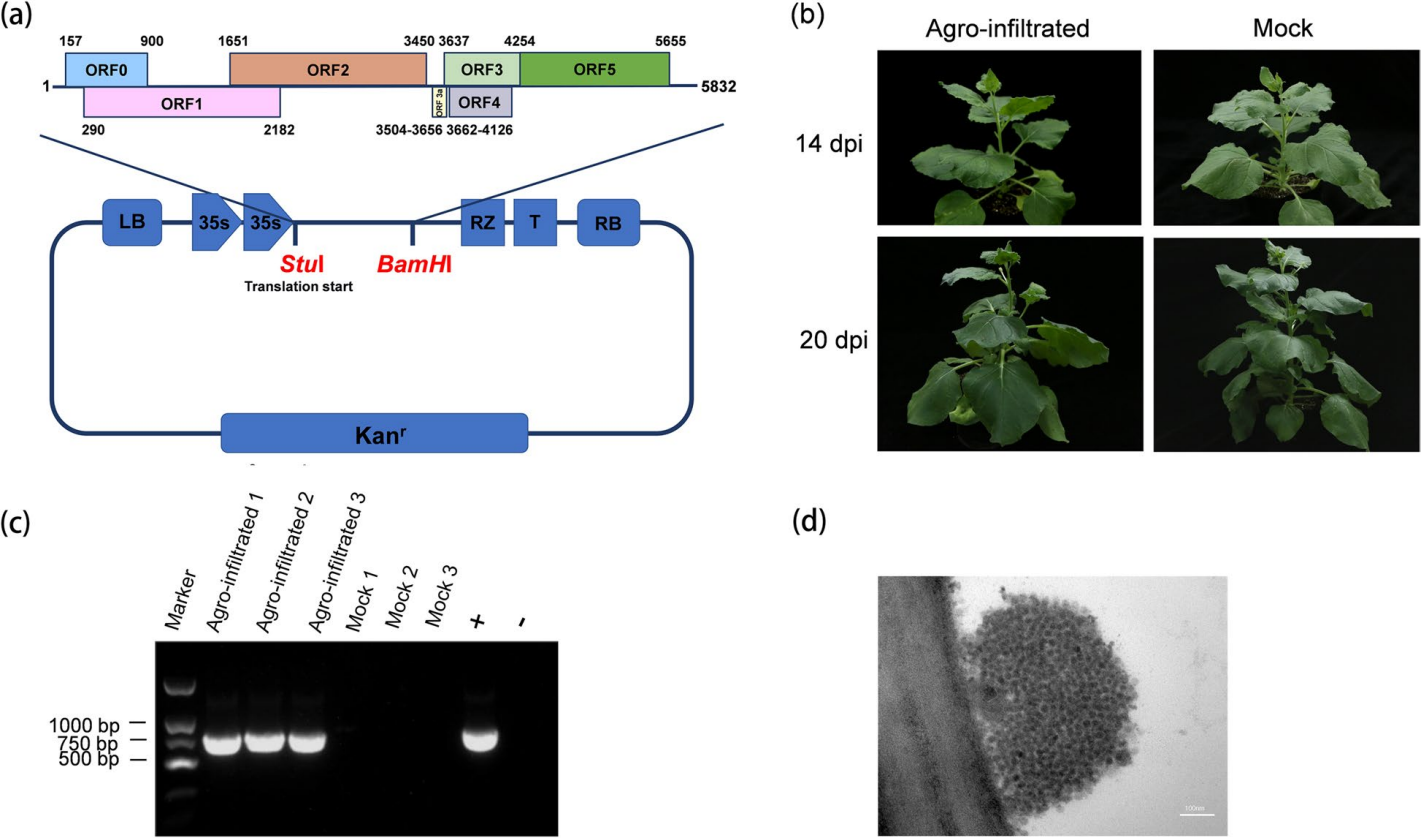
Construction and agroinoculation of an infectious cDNA clone of RDPV. **a** Structural schematic diagram of the full-length infectious cDNA clone of RDPV. The full-length genomic DNA of RDPV was amplified via RT‒PCR and subcloned and inserted into the pCass4-RZ vector between the *Stu* I and *BamH* I sites. **b** Symptoms of *N. benthamiana* plants inoculated with the infectious clone of RDPV (left) and of the mock *N. benthamiana* plant (right) inoculated with the empty pCass4-RZ-RDPV at 14 dpi and 20 dpi. **c** RT‒PCR results of RDPV infection in agroinfiltrated *N. benthamiana* plants at 14 dpi. Mocks 1–3 were three *N. benthamiana* plants inoculated with the empty vector pCass4-RZ-RDPV. + is a known RDPV-infected rice plant used as the positive control, and – is a known uninfected rice plant used as the negative control. **d** Transmission electron microscopy image showing RDPV virions in infected *N. benthamiana* plants inoculated with the infectious cDNA clone of RDPV

The complete genome of RDPV was cloned and inserted into the vector pCass4-RZ to produce an infectious cDNA clone of RDPV, pCass4-RZ-RDPV (Fig. 3a). pCass4-RZ-RDPV was used to transform *A. tumefaciens* strain GV3101, which was subsequently infiltrated into *N. benthamiana* leaves. At 14 dpi, the infiltrated *N. benthamiana* plants did not show obvious virus-like symptoms (Fig. 3b). To investigate RDPV infection, total RNA was extracted from systematic leaves of the tested *N. benthamiana* plants and subjected to RT‒PCR detection using RDPV-specific primer pairs. The RT‒PCR results demonstrated that the infiltrated plants were infected by RDPV (Fig. 3c). Furthermore, transmission electron microscopy (TEM) detected many spherical virus particles approximately 30 nm in size in the infected *N. benthamiana* plant tissues (Fig. 3d). These data indicate that an infectious cDNA clone of RDPV was successfully constructed and that RDPV can infect *N. benthamiana* plants via agroinfiltration. The same rice virus can infect different plants, and even different rice cultivars can cause different symptoms or asymptoms. Thus, we speculate that RDPV cannot induce apparent symptoms in *Nicotiana benthamiana* plants, most likely because *N. benthamiana* is not the natural host of RDPV.

## Discussion

Serological and PCR-based molecular approaches, including dot enzyme-linked immunosorbent assay (dot-ELISA), colloidal gold immunochromatographic test strip, RT‒PCR and real-time quantitative RT‒PCR (RT‒qPCR), are frequently applied to detect plant virus infection in both plants and insect vectors [20–23]. However, these monitoring approaches require prior knowledge of viral genomic and protein information and are thus only suitable for known virus detection but not for unknown novel virus detection. Fortunately, the high-throughput RNA sequencing (RNA-seq) technique was successfully established. It can simultaneously sequence millions or even tens of millions of DNAs and does not require knowledge of the genomic characteristics of the virus in advance [10]. Thus, it has been widely used to identify novel viruses and enriches virus resources in the last decade [10–12]. In this work, we discovered a novel polerovirus tentatively named rice dwarf polerovirus (RDPV) via RNA-seq in rice plants showing dwarfing and fewer tillering symptoms collected from paddy fields in Hainan Province, China. The RDPV genome consists of a 5832 nt positive, single-stranded RNA molecule deduced to encode seven viral proteins. Phylogenetic analyses revealed that RDPV fell within an evolutionary branch with known viruses in the *Polerovirus* genus. Furthermore, we constructed an infectious cDNA clone of RDPV and found that it can infect *N. benthamiana* plants. To our knowledge, RDPV is the first polerovirus that infects rice plants in paddy fields. These findings contribute to expanding our knowledge of rice polerovirus and its vector diversity and the development of efficient strategies to manage this rice viral disease.

Most plant viruses depend on insect vectors for field transmission. The interactions between viruses and insect vectors are highly specific; thus, only one vector or a group of vectors from the same family is able to transmit a given virus [20, 21]. Poleroviruses are phloem-restricted RNA plant viruses that are exclusively transmitted by aphids [9]. However, Ghosh and colleagues [8] recently reported a new pepper polerovirus named pepper whitefly borne vein yellow virus (PeWBVYV), which is transmitted by the whitefly *Bemisia tabaci* but not aphids. Interestingly, our virus transmission experiments demonstrated that both *B. formosana* Takahash and *R. padi* insects can recover RDPV and transmit RDPV from infected rice plants to healthy rice seedlings, but aphids *S. avenae* and *S. graminum* cannot. Thus, we concluded that *B. formosana* Takahash and *R. padi* are two insect vectors of RDPV. Surprisingly, RDPV can be transmitted by both whitefly and aphid vectors, which has never been reported before. This is the first time that a plant virus was found to be transmitted by two completely different vectors, whiteflies and aphids. In addition, both *B. formosana* Takahash and *R. padi* are important harmful insects in the field, and *R. padi* mainly harms wheat plants, whereas *B. formosana* Takahash is a common whitefly in rice fields in China. Thus, we speculate that *B. formosana* Takahash is the primary vector of RDPV in paddy fields. The first whitefly-borne rice virus and its whitefly vector identified in this study will facilitate the elucidation of RDPV epidemic laws in the field and help control this rice viral disease.

## Conclusions and perspectives

In this work, we identified a novel rice polerovirus tentatively named rice dwarf polerovirus (RDPV) via RNA-seq on field-collected rice plants with dwarfing and fewer tillering symptoms. The RDPV genome is a positive, single-stranded RNA molecule that is 5832 nt in size. RDPVs fall on an evolutionary branch with other known viruses in the *Polerovirus* genus in phylogenetic trees. Virus transmission experiments demonstrated that RDPV can be transmitted by two completely different insect vectors, i.e., whiteflies and aphids. In addition, an infectious cDNA clone of RDPV was constructed and can infect *N. benthamiana* plants via agroinfiltration. Taken together, RDPV is the first novel rice polerovirus that infects rice plants in the field. Our findings contribute to the analysis of the genomic function of RDPV and the development of efficient strategies to manage this rice viral disease.

## Materials and methods

### Plant material, plant growth, and aphid vectors

In the spring of 2023, rice plants with dwarfing and fewer tillering symptoms were discovered in paddy fields in Sanya City, Hainan Province, China, and collected to identify viral agents. Rice and *Nicotiana benthamiana* plants were grown in a glasshouse maintained at 28 °C/22 °C (day/night) with a 16 h/8 h (light/dark) photoperiod.

Aphids (*R. padi*, *S. avenae* and *S. graminum*) and rice whitefly (*B. formosana* Takahashi) vectors were reared on healthy rice seedlings in large plastic cups covered with insect-proof mesh at 24 °C. In April 2024, 21 rice plants with dwarfing symptoms were separately collected from 21 rice fields in Sanya City and Lingshui County in Hainan Province, China, to investigate the infection rate of RDPV in paddy fields.

### RNA-seq and de novo assembly

The collected rice plants were ground into a fine powder in liquid nitrogen and subjected to total RNA extraction via the EASYspin Plus Complex Plant RNA Kit (Aidlab Biotech, Beijing, China) following the manufacturer’s instructions. After removing ribosomal RNAs, a cDNA library was constructed via the TruSeq RNA Sample Prep Kit (Illumina, San Diego, CA, USA) as instructed, followed by sequencing on the Illumina HiSeq X-ten platform. The low-quality reads were deleted via CLC Genomics Workbench 9.5 software (Qiagen, Valencia, CA, USA), and the high-quality reads were de novo assembled via the Trinity v2.3.2 program.

### RT‒PCR, 5’/3’ rapid amplification of cDNA ends (5’/3’ RACEs)

The collected rice plants or insects were ground into a fine powder in liquid nitrogen and subjected to total RNA extraction via TRIzol (Invitrogen, Carlsbad, CA, USA) as instructed. For RT‒PCR amplification of the viral genomic segments, cDNA was synthesized via M-MLV (TaKaRa, Tokyo, Japan) and specific reverse primers and amplified via 2 × Phanta DNA polymerase (Vazyme, Nanjing, China) following the manufacturer’s instructions. To obtain the 5’ and 3’ terminal sequences of the RDPV genome, 5’/3’ RACE was performed via the HiScript-TS 5’/3’ RACE Kit (Vazyme, Nanjing, China) as instructed. The resulting PCR products were subjected to Sanger sequencing. All primers used in this work are listed in Table S1.

### Genome organization, phylogenetic and sequence identity analyses of RDPV

The ORFs of RDPV were predicted through the NCBI ORFfinder service (http://www.ncbi.nlm.nih.gov/orffinder) and with the potato leafroll virus (PLRV) genome (GenBank accession no. MK116549) as a model. Domains of viral proteins were predicted via the Conserved Domain Search Service (CD-Search) software in the NCBI (https://www.ncbi.nlm.nih.gov/Structure/cdd/wrpsb.cgi). To elucidate the evolutionary relationship of RDPV, sequences of representative viruses from the *Solemovirideae* and *Tombusviridae* families were retrieved from the GenBank database (Supplementary Table S2). The genomic sequences and RdRp and CP aa sequences of these viruses were aligned via ClustalW in MEGA X software (https://www.megasoftware.net/), as described previously [11]. Phylogenetic analysis was performed via the maximum-likelihood method in MEGA X with 1000 bootstrap replicates (https://www.megasoftware.net/).

### Construction and agroinfiltration of the RDPV infectious cDNA clone

The full-length genomic cDNA of RDPV was cloned and inserted into the pCass4-RZ plasmid [9] to produce the pCass4-RZ-RDPV infectious clone, which was subsequently used to transform *Escherichia coli* DH5α and *Agrobacterium tumefaciens* GV3101. The cultured agrobacteria harboring the pCass4-RZ-RDPV infectious clone were diluted to OD_600_ = 1.0 with infiltration buffer (10 mM MgCl_2_, 10 mM MES and 100 μM acetosyringone, pH 5.8) and infiltrated into *N. benthamiana* leaves with a needle-less syringe. The infiltrated *N. benthamiana* plants in the author’s greenhouse were continuously observed and photographed.

### Virus inoculation by insect transmission

RDPV-free *B. formosana* Takahashi plants were captured in a paddy field in Hangzhou City of Zhejiang Province, China, and identified in the author’s laboratory. The RDPV-free aphids *R. padi*, *S. avenae,* and *S. graminum* were provided by Dr. Julian Chen (Institute of Plant Protection, Chinese Academy of Agricultural Sciences). Rice whiteflies and aphids were reared separately on healthy rice seedlings. For virus inoculation, nonviruliferous vectors were collected from healthy rice seedlings with a brush and starved for 2 h before being fed on RDPV-infected rice plants [9]. At 3 days after feeding, rice whiteflies or aphids were collected from infected rice plants and transferred to healthy rice seedlings (three vectors/rice seedling). At 7 days after feeding, the vectors were killed with a pesticide, and the inoculated rice seedlings were planted in the author’s greenhouse. Virus-free rice whiteflies and virus-free aphids from healthy rice plants were used as negative controls.

### Transmission electron microscopy

For ultrathin section observation, the rice plant tissues or stem tissues of *N. benthamiana* plants were cut into 1 mm × 3 mm pieces and fixed in 2.5% glutaraldehyde overnight and then in 1% osmium tetroxide. Each fixation was followed by a 15 min rinse with 0.1 M sodium phosphate buffer (pH 7.0) three times to remove the fixative. Then, the fixed samples were dehydrated with a graded ethanol series (50%, 70%, 80%, 90%, 95%, and 100%) followed by 100% acetone for 20 min each at room temperature. The samples were embedded in Spurr resin (SPI-EM, West Chester, USA) and solidified at 70 °C for 48 h [24]. Untrathin sections (70–100 nm) of the sample blocks were cut with a UC7 microtome (Leica, Vienna, Austria) with a diamond knife (Diatome, Switzerland) and mounted onto Formvar-coated grids. After being stained with 2% uranyl acetate for 10 min followed by 1% lead citrate for 10 min, the sample sections were observed via an H-7650 transmission electron microscope (TEM) (Hitachi, Ibaraki, Japan) at 80 kV and photographed with a Gatan 832 CCD camera (Gatan, Pleasanto, CA, USA).

## Supplementary Information

Supplementary Material 1: Table S1. Primers used in this work. Table S2. Viral genomic sequences used in this work. Table S3. Identity percentage of nucleotide and protein sequences of RDPV with known numbers in the genus *Polerovirus* sequences. Figure S1. RT‒PCR analysis of RDPV infection in *Sitobion avenae*, *Schizaphis graminum* and *Rhopalosiphum padi* fed RDPV-infected rice plants for 3 days following 7 days of feeding on healthy rice seedlings.

## Data Availability

Not applicable.
