# Supplementary material for: Discovery of a novel whitefly- and aphid-transmitted polerovirus on rice plants with dwarfing and fewer tillering symptoms
Source: Crop Health. 2024 Aug 6;2(1):13. doi: 10.1007/s44297-024-00033-0 (PMC12825964; doi:10.1007/s44297-024-00033-0)
Supplement: Supplementary file 1 — Supplementary Material 1: Table S1. Primers used in this work. Table S2. Viral genomic sequences used in this work. Table S3. Identity percentage of nucleotide and protein sequences of RDPV with known numbers in the genus Polerovirus sequences. Figure S1. RT‒PCR analysis of RDPV infection in Sitobion avenae, Schizaphis graminum and Rhopalosiphum padi fed RDPV-infected rice plants for 3 days following 7 days of feeding on healthy rice seedlings. [file 44297_2024_33_MOESM1_ESM.zip › RDPV Supplemental tables_ESM.docx]

Supplemental tables

| **Table 1: Primers used in this work** | | |
| --- | --- | --- |
|  |  |  |
| **Application** | **Name** | **Sequence (5' to 3')** |
| Primers used for RT-PCR detection | RDPV-F | ACATCTTCAACGCTCACTTC |
|  | RDPV-R | TCCTCACCGGTGGTTTCAAT |
| Primers used for 3’/5’RACE | RDPV-3'GSP-1 | AATTAGGCTCTGCCCACGCGAGAGAAGC |
|  | RDPV-3'GSP-2 | GGAGACCGCATGGCTGTTGATGAGC |
|  | RDPV-5'GSP-1 | TCGCAAATTAAGTTCCATGCACGCGCGG |
|  | RDPV-5'GSP-2 | GGCCCAGTCCCAAAAATCCACTTGAACG |
| Primers used for PCR amplification for vector construction | pCass4-RZ-RDPV-F | tttcatttggagaggACAAAAGAAATCCAGGCAGAG |
|  | pCass4-RZ-RDPV-R | tgacagggtatcggatcAACCAGAGTTCCGAAGAAG |
| F, forward primer; R, reverse primer; adaptor sequence was added for vector construction | | |

| **Table 2: Viral genomic sequences used in this work** | | | |
| --- | --- | --- | --- |
|  |  |  |  |
| **Family** | **Genus** | **virus** | **GenBank Accession No.** |
| ***Solemoviridae*** | ***polerovirus*** | African eggplant yellowing virus | KX856972 |
|  |  | Barley virus G | KT962089 |
|  |  | Beet chlorosis virus | NC_002766.1 |
|  |  | Beet mild yellowing virus | NC_003491.1 |
|  |  | Beet western yellows virus | NC_004756.1 |
|  |  | Brassica yellows virus | HQ388348 |
|  |  | Cardamom polerovirus | BK013145 |
|  |  | Carrot polerovirus 2 | OP886451.1 |
|  |  | Carrot red leaf virus | NC_006265.1 |
|  |  | Cereal yellow dwarf virus RPS | NC_002198.2 |
|  |  | Cereal yellow dwarf virus RPV | NC_004751.1 |
|  |  | Chickpea chlorotic stunt virus | NC_008249.1 |
|  |  | Cotton leafroll dwarf virus | NC_014545.1 |
|  |  | Cowpea polerovirus 1 | KY364846 |
|  |  | Cucurbit aphid-borne yellows virus | NC_003688.1 |
|  |  | Faba bean polerovirus 1 | MH464873 |
|  |  | Grapevine polerovirus 1 | LC507098 |
|  |  | Hemisteptia virus A | ON416859 |
|  |  | Maize yellow mosaic virus | MK652150 |
|  |  | Melon aphid-borne yellows virus | NC_010809.1 |
|  |  | Panicum distortion mosaic virus | LC424839.1 |
|  |  | Pepo aphid-borne yellows virus | NC_030225.1 |
|  |  | Pepper leafroll virus | LT220496 |
|  |  | Potato leafroll virus | MK116549.1 |
|  |  | Pumpkin polerovirus | MG800833.2 |
|  |  | Soybean chlorotic leafroll virus | OM507197 |
|  |  | Suakwa aphid-borne yellows virus | YP_006666509.1 |
|  |  | Sugarcane yellow leaf virus | NC_000874.1 |
|  |  | Tobacco vein distorting virus | NC_010732.1 |
|  |  | Triticum yellow stripe virus | OM829809.1 |
|  |  | Turnip yellows virus | NC_003743.1 |
|  |  | Wheat leaf yellowing-associated virus | NC_035451.1 |
|  | ***Enamovirus*** | Pea enation mosaic virus 1 | L04573 |
| ***Tombusviridae*** | ***Tombusvirus*** | Grapevine Algerian latent virus | NC_011535.1 |
|  |  | Tomato bushy stunt virus | NC_001554.1 |

| **Table 3: Identity percentage of nucleotide and proteins sequence of RPDV with known numbers in the genus *Polerovirus*** | | | | | | | | |
| --- | --- | --- | --- | --- | --- | --- | --- | --- |
|  |  |  |  |  |  |  |  |  |
| **Virus name** | **Nucleotide Identities** (%) | **Amino Acids Identities (%)** | | | | | | |
|  |  | **ORF0** | **ORF1** | **ORF1-2 fusion** | **ORF3a** | **ORF3** | **ORF4** | **ORF 3-5 fusion** |
| Panicum distortion mosaic virus | 77.9 | 62.8 | 70.2 | 78.6 | - | 87.8 | 87.6 | 76.1 |
| Triticum yellow stripe virus | 63.5 | 27.2 | 44.4 | 58.0 | - | 64.2 | 73.2 | 57.1 |
| Cereal yellow dwarf virus RPS | 62.5 | 35.3 | 44.5 | 46.3 | - | 66.7 | 52.0 | 57.9 |
| Cereal yellow dwarf virus RPV | 61.5 | 15.7 | 32.5 | 59.7 | - | 61.8 | 47.6 | 49.3 |
| Wheat leaf yellowing-associated virus | 54.8 | 11.9 | 31.0 | 46.5 | 50.0 | 44.9 | 39.9 | 32.0 |
| Potato leafroll virus | 53.5 | 22.1 | 30.6 | 19.6 | 68.9 | 65.3 | 42.2 | 33.6 |
| Carrot polerovirus 2 | 52.9 | 33.3 | 32.5 | 46.6 | 53.3 | 45.5 | 33.8 | 42.2 |
| Beet mild yellowing virus | 51.7 | 19.3 | 31.4 | 41.0 | - | 60.8 | 47.0 | 45.6 |
| Beet western yellows virus | 51.6 | 17.9 | 10.3 | 44.0 | - | 59.3 | 45.0 | 45.1 |
| Turnip yellows virus | 51.3 | 18.3 | 32.5 | 45.3 | 64.4 | 59.3 | 47.7 | 49.3 |
| Carrot red leaf virus | 50.6 | 14.2 | 32.2 | 48.3 | 57.8 | 46.5 | 31.6 | 37.0 |
| Beet chlorosis virus | 49.9 | 17.1 | 29.4 | 40.8 | - | 59.3 | 44.3 | 45.8 |
| Maize yellow mosaic virus | 48.6 | 16.4 | 32.8 | 44.4 | 47.7 | 58.4 | 36.3 | 35.5 |
| Melon aphid-borne yellows virus | 48.5 | 17.8 | 32.2 | 47.0 | 56.2 | 58.6 | 41.9 | 34.6 |
| Suakwa aphid-borne yellows virus | 48.3 | 13.0 | 25.3 | 46.8 | - | 60.1 | 35.0 | 34.0 |
| Cucurbit aphid-borne yellows virus | 48.1 | 18.5 | 33.0 | 45.4 | 68.9 | 55.2 | 39.9 | 36.0 |
| Chickpea chlorotic stunt virus | 47.8 | 15.6 | 27.9 | 59.0 | 57.9 | 47.5 | 31.6 | 37.3 |
| Tobacco vein distorting virus | 47.8 | 22.8 | 30.5 | 56.2 | 60.0 | 54.0 | 43.3 | 44.3 |
| Pepo aphid-borne yellows virus | 46.6 | 12.9 | 24.9 | 43.0 | 62.2 | 56.1 | 39.5 | 34.5 |
| Cotton leafroll dwarf virus | 46.3 | 16.5 | 32.2 | 45.2 | 62.2 | 60.1 | 44.2 | 35.7 |
| Pumpkin polerovirus | 46.1 | 14.2 | 26.4 | 58.6 | 62.2 | 56.1 | 43.9 | 34.3 |
| Sugarcane yellow leaf virus | 45.7 | 13.3 | 30.4 | 39.8 | - | 43.1 | 38.8 | 25.1 |

- viral sequences are not available. Red numbers indicate the highest identity.
