# Supplementary figures and images for: Discovery of a novel whitefly- and aphid-transmitted polerovirus on rice plants with dwarfing and fewer tillering symptoms
Source: Crop Health. 2024 Aug 6;2(1):13. doi: 10.1007/s44297-024-00033-0 (PMC12825964; doi:10.1007/s44297-024-00033-0)

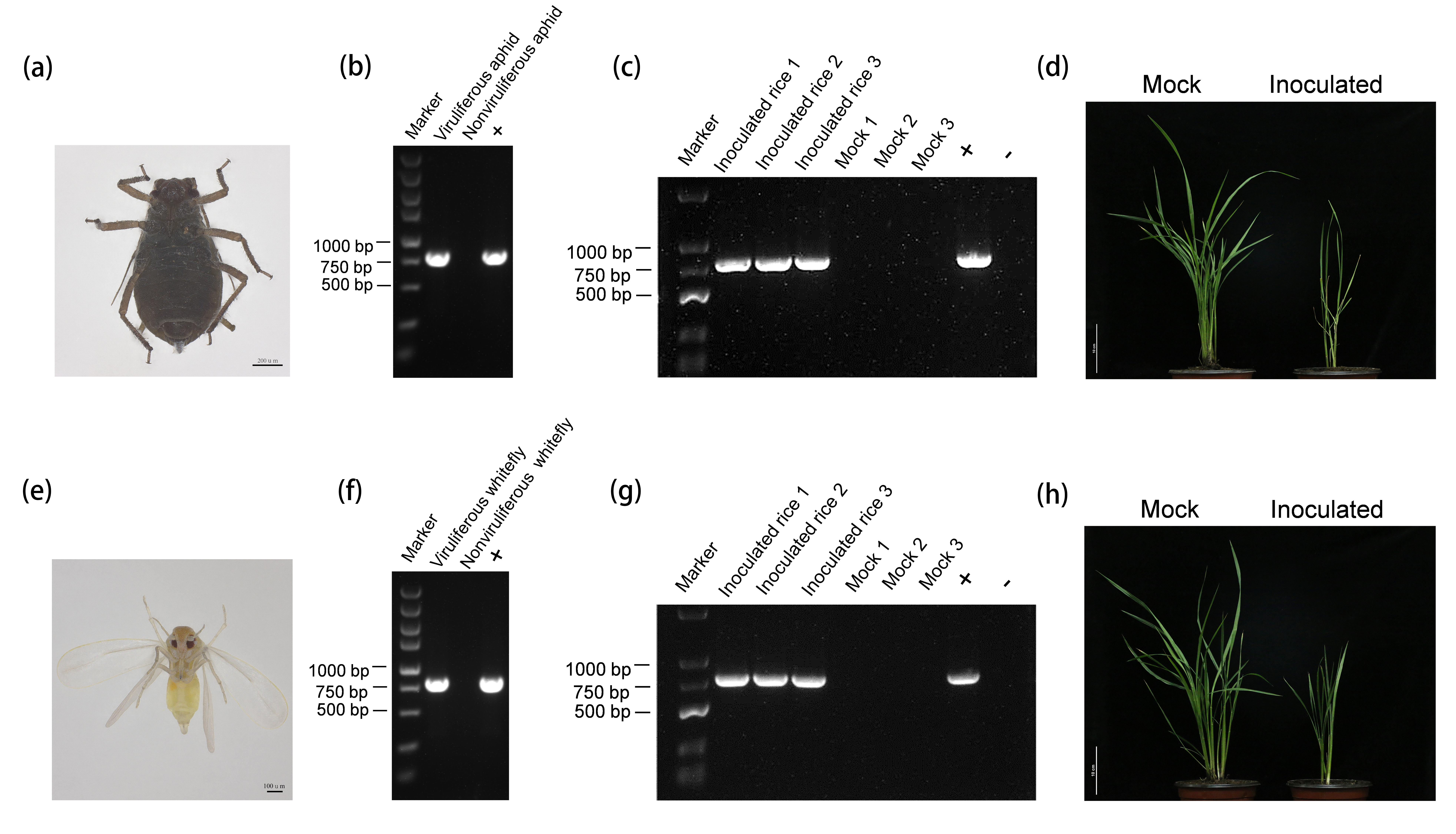

Supplement: Supplementary file 1 — Supplementary Material 1: Table S1. Primers used in this work. Table S2. Viral genomic sequences used in this work. Table S3. Identity percentage of nucleotide and protein sequences of RDPV with known numbers in the genus Polerovirus sequences. Figure S1. RT‒PCR analysis of RDPV infection in Sitobion avenae, Schizaphis graminum and Rhopalosiphum padi fed RDPV-infected rice plants for 3 days following 7 days of feeding on healthy rice seedlings. [file 44297_2024_33_MOESM1_ESM.zip › Fig. 2_ESM.tif]
